# Identification of a Novel Inhibitory Allosteric Site in p38α

**DOI:** 10.1371/journal.pone.0167379

**Published:** 2016-11-29

**Authors:** Patricia Gomez-Gutierrez, Pedro M. Campos, Miguel Vega, Juan J. Perez

**Affiliations:** 1 Allinky Biopharma. Madrid Scientific Park. Faraday, 7. Campus de Cantoblanco, Spain; 2 Dept. of Chemical Engineering. Universitat Politecnica de Catalunya. ETSEIB. Av. Diagonal, Spain; UMR-S1134, INSERM, Université Paris Diderot, INTS, FRANCE

## Abstract

In the present study, we report the discovery of a novel allosteric inhibitory site for p38α, a subclass of the mitogen-activated protein kinases (MAPK) family. The putative site was discovered after inspection of the crystallographic structure of the p38α-MK2 complex. MK2 (MAPK-activated protein kinase 2) is an interesting protein playing a dual role as modulator and substrate of p38α. This intriguing behavior is due to the ability of the two proteins to form distinctive heterodimers when p38α is phosphorylated or not. We hypothesized that the regulatory action of MK2 is due to its capability to keep p38α in an inactive conformation and consequently, we investigated the atomic structure of the p38α-MK2 complex to understand such regulatory behavior at the molecular level. After inspection of the complex structure, two peptides designed from the MK2 regulatory loop in contact with p38α with sequences Tyr^1^-Ser^2^-Asn^3^-His^4^-Gly^5^-Leu^6^ (peptide-1) and [Phe^0^]-peptide-1 (peptide-2) in their zwitterionic form were investigated for their phosphorylation inhibitory capability *in vitro*. Since both peptides exhibited inhibitory capability of the p38α kinase mediated phosphorylation of MEF2A, in a subsequent step we pursued the discovery of small molecule peptidomimetics. For this purpose we characterized in detail the peptide-p38α interaction using molecular dynamics simulations, leading to the definition of a pharmacophore for the peptide-protein interaction. This hypothesis was used as query for a *in silico* screening, leading to the discovery of a fused ring compound with micromolar inhibitory activity. Site-directed mutagenesis studies support that the compound binds to the putative novel allosteric site in p38α.

## Introduction

Protein phosphorylation is the most widespread post-translational modification used in signal transduction. This process is catalyzed by protein kinases, a large family of highly related enzymes covering about 2% of the human genome [1]. Protein phosphorylation involves the transfer of the γ-phosphate group of ATP onto specific amino acids that exhibit a free hydroxyl group in substrate proteins and peptides, with a concomitant conformational change in the structure of the substrates causing them to become activated or deactivated. This process plays a central role in the regulation of a wide array of signaling pathways that control metabolism, cell cycle progression and cell proliferation, death, differentiation and survival [2].

Dysregulation of kinase activity can result in dramatic changes directly affecting the control of the above mentioned processes, being responsible for the onset and/or progression of various human diseases including inflammatory, cardiovascular, metabolic, neurodegenerative and cancer [3]. Accordingly, protein kinases are considered important targets for therapeutic intervention. At the present there are more than thirty kinase inhibitors approved and more than a hundred in clinical trials, in addition to those in a preclinical state [4]. The first kinase inhibitors discovered (type I) targeted the catalytic ATP binding site. However, due to the high level of similarity of this site across family members, it has been difficult to achieve the required pharmacological selectivity, mainly for the treatment of non-life-threatening diseases like many immunological dysfunctions. For this reason, interest has moved recently to the discovery of allosteric inhibitors aimed at exploiting structural features and regulatory mechanisms that are unique to a particular kinase.

In contrast to type I kinase inhibitors, allosteric inhibitors induce a redistribution of the kinase conformational ensemble, increasing the population of inactive conformations through the displacement of specific motifs that are key for the catalytic activity from their optimal positions. Moreover, unlike the specific requirements of the active conformation responding to a set of highly conserved features, impairing activation allosterically can be done in diverse ways. Thus, type II and III kinase inhibitors bind to allosteric subsites next to the ATP binding site that emerge through the acquisition of the inactive conformations DFG-out and αC-out. Interestingly, in contrast to the latter, the former type of inhibitors are ATP competitive. Some of the allosteric inhibitors described in the literature are exceptionally selective while others are not, stressing the point that allosteric inhibitors are not necessarily selective. Actually, inhibitors that bind to remote areas from the ATP-binding site typically present a great degree of selectivity, due to the fact that these sites are generally less conserved in sequence and structure. This kind of inhibitors are known as type IV kinase inhibitors, and they mainly exert their function allosterically by stabilizing inactive conformations or through the blockade of interactions with other proteins [5].

**Fig 1:**
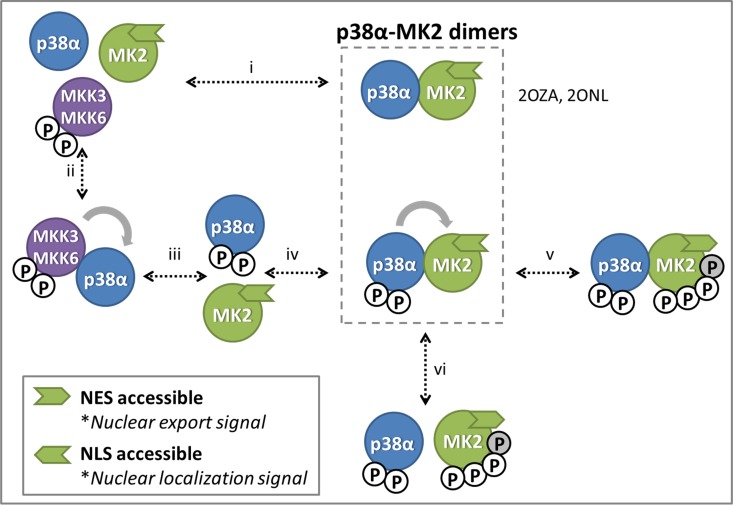
Scheme of the interaction between p38α and MK2 as proposed by teer Haar et al. [11]. (i) In the nucleus of the cell p38α and MK2 can form a heterodimer that prevents both proteins to phosphorylate their respective substrates. Crystal structures with PDB codes 2OZA [10] and 2ONL [11] may constitute representative structures of this heterodimer present in the nucleus. (ii) Alternatively p38α can interact with MKK3 or MKK6 (iii) to be phosphorylated by the action of them. (iv) Once p38α is phosphorylated on residues T180 and Y182, it can form an alternative heterodimer with MK2, (vi) which will induce phosphorylation of MK2 residues T25, T222, T272 and T334 by p38α. Phosphorylation of MK2 in residue T334 (grey phosphate) induces a conformational change that makes its nuclear export signal (NES) accessible, permitting the p38-MK2 heterodimer to be translocated to the cytoplasm, where they can phosphorylate diverse substrates. V) Alternatively, the dimer can dissociate in the nucleus and both proteins will phosphorylate diverse substrates until a phosphatase dephosphorylates them.

In the present study we report the discovery of a novel allosteric inhibitory site for p38α, a subclass of the mitogen-activated protein kinases (MAPK) family. These enzymes respond to stress stimuli such as ultraviolet irradiation, heat or osmotic shock, as well as to numerous extracellular mediators of inflammation, resulting in a variety of adaptive and physiological responses, including cell differentiation, apoptosis and autophagy. These actions are mediated by phosphorylation of diverse transcription factors, elongation factors and downstream kinases [6]. Among the diverse p38α substrates, MAPK-activated protein kinase 2 (MK2) plays a dual role as modulator and substrate [7] as shown pictorially in Fig 1. This intriguing behavior is due to the ability of the two proteins to form distinctive heterodimers when p38α is phosphorylated or not. Thus, in the nucleus when the two proteins are unphosphorylated, they form a high affinity complex (K_D_ = 2.5 nM) where the ATP-binding sites of both kinases are buried in the heterodimer interface, preventing them from phosphorylating their respective substrates [8]. Cell stress provokes the phosphorylation of p38α by activated MKK3 or MKK6 with the subsequent dissociation of the heterodimer. In turn, activated p38α phosphorylates MK2 as well as a series of transcription factors in the nucleus, activating gene expression. Phosphorylated p38α can also form a heterodimer with MK2 that exhibits a reduced stability (K_D_ = 60 nM). This heterodimer can be exported to the cytoplasm, where each of the kinases may phosphorylate diverse substrates until they are deactivated by phosphatases [9]. We hypothesized that the regulatory action of MK2 is due to its ability to keep p38α in its inactive conformation and consequently, we investigated the atomic structure of the p38α-MK2 heterodimer to understand the details of such inhibitory behavior at the molecular level.

There are two available crystallographic structures of the complex p38α-MK2 with both unphosphorylated kinases at different resolution (pdb entries 2OZA [10] and 2ONL [11]). The structure of the complex reveals five discontinuous contact regions between the two proteins. Closer inspection of the complex prompted us to investigate the contact region involving the regulatory phosphorylation region of p38α comprising the segment Gly^173^-Tyr^188^, bound to a regulatory phosphorylation region of MK2 comprising residues Tyr^264^-Tyr^284^. In this contact region the two segments are stabilized by diverse intermolecular hydrogen bonds forming a short antiparallel β-sheet [10]. Moreover, since the activation loop of p38α is found in a conformation that differs from those observed in other crystallographic structures, we hypothesized that interaction between the two protein segments induces the p38α activation loop to adopt an inactive conformation. Previous results published in the literature partially support present hypothesis. Actually, a peptide substrate binds to the same regulatory phosphorylation region of the insulin kinase domain as it is proposed in the present work, forcing the activation loop to adopt a folded conformation [12]. Moreover, that structure was used for the design of selective bisubstrate inhibitors that resulted from the combination of a ATP analog with a peptide that binds to this regulatory phosphorylation region [13].

Accordingly, we investigated the *in vitro* inhibitory capability of two peptides designed from the MK2 regulatory loop in contact with p38α with sequences: Phe^1^-Tyr^2^-Ser^3^-Asn^4^-His^5^-Gly^6^-Leu^7^ (peptide-1) and [Phe^0^]-peptide-1 (peptide-2), in their zwitterionic form. We further analyzed the potential of this site for the design of allosteric peptidomimetic inhibitors by means of a detailed study of the structural features of the peptide-p38α interaction, leading to the development of a pharmacophore that was subsequently used for the discovery of small molecule hits by virtual screening.

## Methods

### Computational Studies

**Fig 2:**
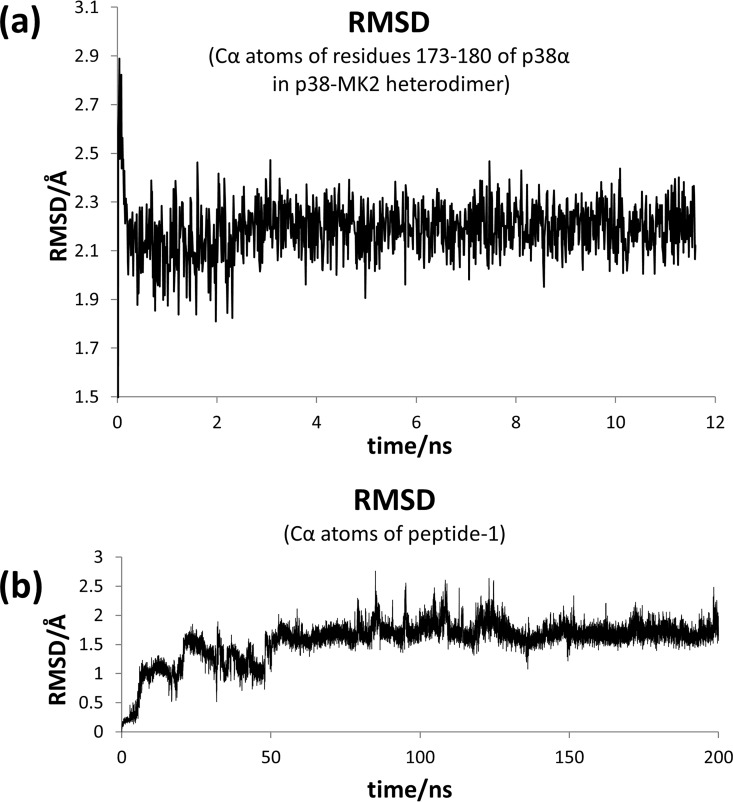
Modeling of the peptide-1-p38α complex. (a) Time evolution of the rmsd of the Cα atoms involved in the segment of the p38α activation loop modelled during the 10ns MD simulation of the dimer. (b) Time evolution of the rmsd of the Cα atoms of peptide-1 along the 200ns MD simulation of the peptide-1-p38α complex.

The atomic coordinates of the p38α-MK2 complex were retrieved from the Protein Data Bank website (PDB ID: 2OZA) [14]. Since the stretch of residues 173–180 of the p38α activation loop is not solved in the crystallographic structure, it was modelled using the Modeller 9v2 program [15]. Specifically, the segment was energy minimized using the steepest descent method followed by a 10ns molecular dynamics (MD) simulation. Sampling began by increasing the temperature of the system steadily up to 300K, followed by an equilibration period consisting of 1ns at a constant pressure and 1ns at a constant volume, followed by a 10ns MD trajectory at constant volume. Time evolution of the Cα atoms root-mean square deviation (rmsd) of the modelled segment in regard to the starting structure shows that after 2.2ns of simulation the structure does not suffer significant variations (Fig 2A). In a subsequent step, the coordinates of the MK2 protein were removed with the exception of the segment 264–269 (peptide-1). This structure was minimized by means of the steepest descent method using a hierarchical procedure in which harmonic positional restrictions were first applied to all backbone atoms to be subsequently released in a second minimization step. At this point the system was submitted to a MD simulation according to the following protocol: first, the temperature of the system was raised to 300K at a rate of 30K/10ps and second, 1ns equilibration at a constant pressure followed by 30ns at a constant volume. Harmonic positional restraints were applied on side chains and backbone atoms and progressively lifted, except for those applied to the backbone atoms of the activation loop that were kept at 0.1 kcal/(mol.Å^2^) during the subsequent 70ns at a constant volume. At this point restraints were lifted and the system was allowed to run for another 100ns. All the simulations were carried out using the program pmemd of the AMBER12 software [16] and the ff99sb force field [17]. The solvent was treated explicitly using the TIP3P water model and periodic boundary conditions were applied using the Particle Mesh Ewald method. A Langevin thermostat was used to control the temperature of the system along the MD simulations, and the Shake algorithm was employed in order to restrict the stretching movement of all bonds involving hydrogen atoms, which allowed the use of a time integration step of 2fs.

Time evolution of the root-mean square deviation (rmsd) of the position of the peptide Cα atoms in regard to its starting structure was performed using Ptraj program of the AMBER12 software [16] and it is shown in Fig 2B. It can be seen that after approximately 60ns of simulation the structure of the peptide is already equilibrated. After the first 100ns, when the restraints are lifted, the rmsd shows a few oscillations to come back to previous values, showing that peptide backbone is not subjected to large structural variations in the sampling process. An analysis of the relevant peptide-protein interactions was performed using the last 40ns of the MD trajectory. Time evolution of the hydrogen bonds was done using the Ptraj program of AMBER12 software, whereas the contribution of each residue to the binding free energy as well as its decomposition in its different contributions was performed using the MMGBSA methodology as implemented in AMBER12 [16]. Finally, the average structure of the last 40ns of the simulation was computed and minimized following the same protocol that was used in the minimization of the initial peptide-1-p38α complex structure.

Analysis of the peptide-protein interactions throughout the MD trajectory permitted to develop a pharmacophore describing the stereochemical recognition requirements that was subsequently used for the discovery of small molecule mimetics of peptide-1 by virtual screening. For this purpose, a lead-like database included in the MOE software (Molecular Operating System) containing around 650,000 commercially available compounds was used for the search [18]. For each compound, in addition to its 3D structure, the database includes a set of conformations generated using a build-up procedure from systematic conformational searches of molecular fragments. Molecules that fulfilled the pharmacophoric hypothesis were subjected to a molecular docking process using the docking module of MOE. Molecules were docked onto the previously obtained average structure of p38α and positioned in the peptide-1 binding pocket guided by pharmacophore requirements, to be subsequently refined by energy minimization leaving the ligand free and permitting limited lateral movement of the side chains of those amino acids within 6 Å of the ligand. Those conformations that after refinement did not fulfill the pharmacophore were discarded. In a subsequent step, a diversity analysis of the remaining molecules was performed [19]. For this purpose molecules were encoded as bit strings using the typed atom triangle (TAT) methodology in which atoms are grouped in trios including information about their chemical nature and mutual distance [20]. In a next step, the distance between bit strings was computed using the Tanimoto coefficient [21]. Finally, molecules were grouped in thirty-one clusters using the Jarvis-Patrick algorithm [22]. For each cluster the molecule with the highest binding energy computed in the docking process was chosen as representative.

### *In vitro* phosphorylation assays

To analyze the inhibitory effect of the peptides and small molecules we performed *in vitro* kinase assays using the ADP-Glo™ system (Promega #V9101). For this purpose, purified recombinant active p38αWT (Proqinase #0443-0000-3) at 10nM was pre-incubated during 10 minutes at 30°C with each of the peptides and small molecules at different concentrations in duplicate to a final volume of 30μl of kinase buffer (Hepes 60mM pH 7.5, MgCl_2_ 3mM, MnCl_2_ 3mM, Sodium Orthovanadate 3mM, DTT 1.2mM). After pre-incubation, p38 peptide substrate (SignalChem #P03-58) and ATP were added to a final concentration of 50μM and 10μM respectively, and then incubated during 40 minutes at 30°C for the kinase reaction. Phosphorylation was measured through the ADP production that was detected with the ADP-Glo™ system and the emitted luminescence was measured with a BMG Fluostar microplate reader.

This process was also repeated to compare the inhibitory capability of compounds to mutants Arg^186^Ala and Arg^189^Ala (10nM) in the conditions previously described, but using inactive p38αWT (Proqinase #0639-0000-1); replacing p38 peptide substrate by ATF2 (Proqinase #0594-0000-2) and adding constitutively active MKK6 (Proqinase #0396-0000-1) as p38 activator. Experiments were carried out in such a way that ADP production due to MKK6 catalysis can be considered as negligible. Moreover, since the activity in p38α mutants is lower than in p38αWT, inhibition was normalized with controls of each mutant without ligand to compensate any effect of the mutation in the kinase activity.

Finally, since the inhibitory action of peptide-1 and -2 resulted negative using the p38α peptide substrate, peptide inhibition experiments were carried out using the ADP Quest™ system. For this purpose, purified active p38αWT (25nM; Invitrogen #PV3304) was pre-incubated with peptide-1 or -2 for 10 minutes at 30°C at different concentrations to a final volume of 40μl of kinase buffer (Hepes 15mM pH 7.4, NaCl 20mM, EGTA 1mM, Tween-20 0.02%, MGCl_2_ 10mM, gamma-globulins 0.1%). After pre-incubation, MEF2A (purified recombinant GST‑tagged full length protein) and ATP were added to a final concentration of 300nM and 100μM respectively, and incubated during 30 minutes at 30°C for the kinase reaction. Phosphorylation was measured through the ADP production that was detected using the ADP Quest™ assay kit (DiscoveRx Corp). Resultant fluorescence was read with a BMG Fluostar microplate reader.

## Results

**Fig 3:**
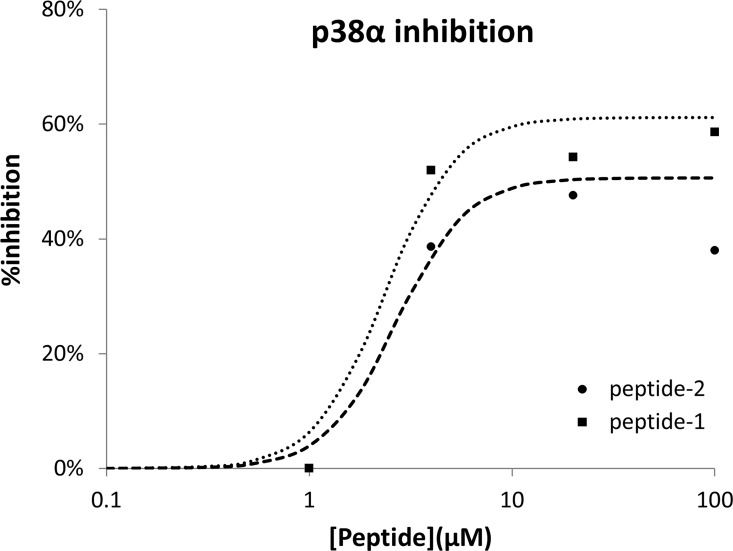
Dose-response curves of the p38α mediated MEF2A phosphorylation inhibition profile exhibited by peptide-1 and peptide-2.

Two fragments of the MK2 regulatory loop in contact with p38α, with sequences: Tyr^1^-Ser^2^-Asn^3^-His^4^-Gly^5^-Leu^6^ (peptide-1) and [Phe^0^]-peptide-1 (peptide-2) in their zwitterionic forms were selected to test their phosphorylation inhibitory capability mediated by p38α *in vitro*. Despite peptides did not show any phosphorylation inhibition capability using the p38α peptide substrate, they exhibited inhibitory capability of the p38α mediated MEF2A phosphorylation in a concentration dependent manner. Fig 3 shows a dose-inhibition curve of p38α kinase mediated phosphorylation of MEF2A, pointing to the capability of both peptides to regulate p38α activity in the micromolar range.

**Fig 4:**
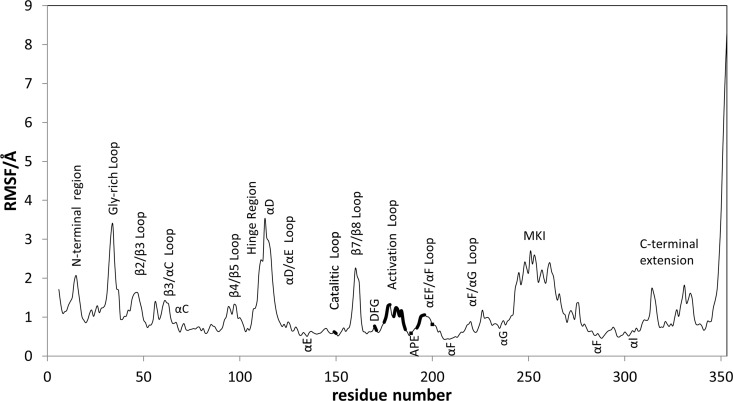
Root-mean square fluctuations (rmsf) of the p38α residues computed using the last 100ns of MD simulation of the peptide-1-p38α complex. Residues involved in the binding site of peptide-1 are highlighted including residues from the catalytic loop as well as from the activation and αEF/αF loops.

In a subsequent step, aimed at discovering small molecule mimics of the peptides with inhibitory capability of p38α, we focused on the characterization of the structural features of the peptide-p38α interaction. For this purpose we run a 200ns molecular dynamics simulation of peptide-1 bound to p38α to allow the peptide to adapt to the receptor. First, since the p38α activation loop is not complete in the crystallographic structure, it was modelled as described in the methods section and its structure refined in the dimer complex environment by means of a 10ns MD simulation. As shown in Fig 2A, the time evolution of the Cα atoms root-mean square deviation (rmsd) of the added segment became stable after the first 2.2ns of the MD trajectory, suggesting that its structure was properly refined. Regarding the peptide-1-p38α complex MD simulation, time evolution of the Cα atoms rmsd of the peptide shows that after approximately 60ns of simulation the structure of the peptide is equilibrated and that the backbone is not subjected to large structural variations (Fig 2B). Root-mean square fluctuation analysis (rmsf) of the protein residues in the peptide-1-p38α complex is depicted pictorially in Fig 4. In agreement with previous dynamic studies of p38α bound to type IIA inhibitors, the profile exhibits higher fluctuations affecting residues located in loops rather than in other structured parts of the protein [23]. Inspection of Fig 4 indicates that residues located in the binding regulatory phosphorylation region of peptide-1 exhibit low fluctuations, ranging from 0.6 to 1.3Å. Interestingly, some regions of the kinase seem to be more flexible than when p38α is bound to a type IIA inhibitor [23]. Specifically, these include residues of the hinge region, which is part of the binding site of ATP-competitive inhibitors; the glycine-rich loop; and two regions that contribute to the formation of the docking groove and ED site: β7/β8 loop and αD helix. These differences in protein flexibility suggest a differential inhibitory mechanism between peptide-1 and type IIA inhibitors.

**Fig 5:**
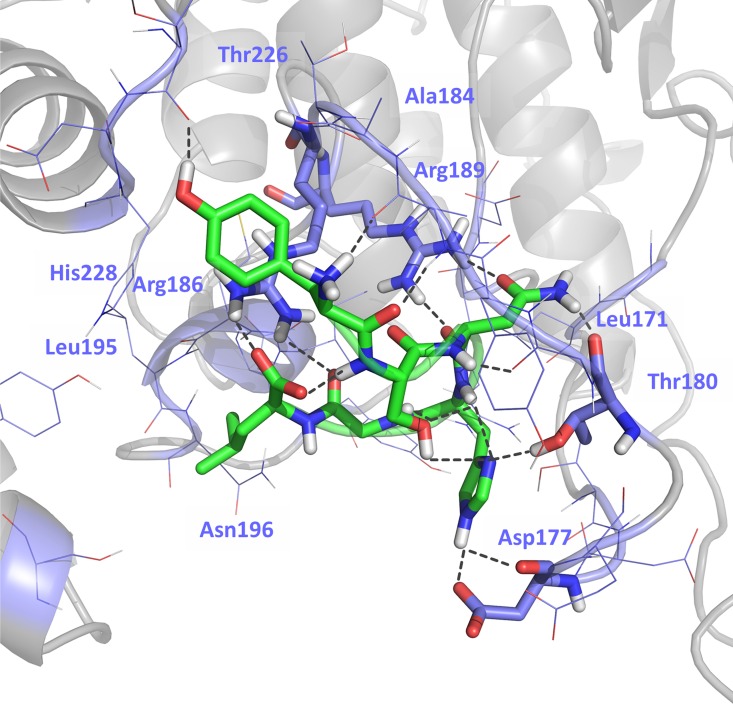
Energy minimized average structure obtained from the last 40ns of the molecular dynamics simulation of peptide-1 bound to the novel allosteric site of p38α proposed in the present work. Regions of p38α involved in the interaction are labelled in light blue.

Since the conformation of the peptide remains stable along the MD run, we computed the minimized average structure of the last 40ns of the MD simulation and used as the bound conformation of peptide-1 to p38α (Fig 5). In the bound conformation the peptide adopts a hairpin conformation stabilized by five intramolecular hydrogen bonds including a triple interaction of the hydroxyl group of Ser^2^ with the amide nitrogen of Gly^5^, the nitrogen-delta of the imidazole ring of His^4^ and the amide nitrogen of the same residue; an interaction between the nitrogen-delta of the imidazole ring and the amide nitrogen of His^4^; and an interaction between one of the oxygen atoms of the carboxyl terminal group of Leu^6^ and the amide hydrogen of Ser^2^.

Analysis of the hydrogen bond interactions between peptide-1 and p38α along the molecular dynamics trajectory reveals five relevant interactions as shown in Fig 5. One involves the carbonyl oxygen of the side chain of peptide-1 residue Asn^3^ with the polar hydrogens of the Arg^189^ guanidine group in p38α, which also interacts with the oxygen carbonyl atom of the peptide-1 Asn^3^ backbone. On the other hand, the hydrogens of the amide group located on the side chain of peptide-1 Asn^3^ interact with the carbonyl oxygen of p38α Thr^180^ backbone. Moreover, a fourth interaction involves the nitrogen-epsilon of the imidazole ring of peptide-1 His^4^ side chain with the carbonyl oxygen of p38α Asp^177^ backbone and the carboxylic atoms of its side chain. Finally, there is a hydrogen bond between the carboxylate oxygens of Leu^6^ and the polar hydrogens of p38α Arg^186^ guanidine group.

**Fig 6:**
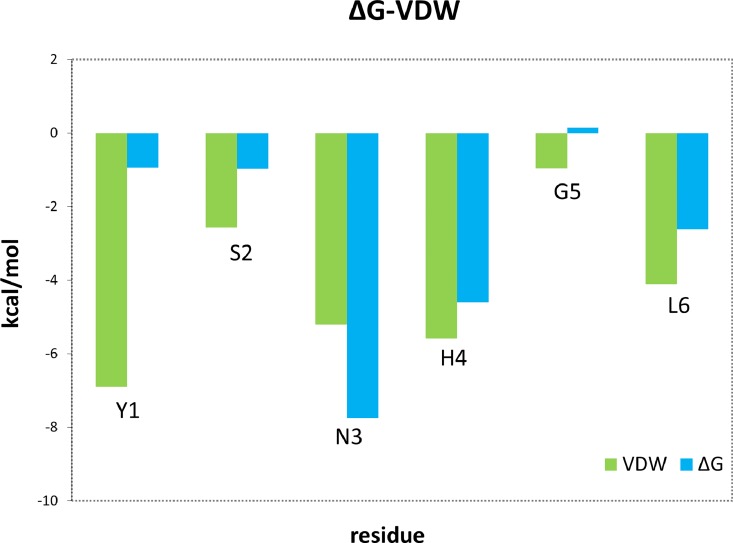
Peptide-1 binding free energy and van der Waals contribution to the interaction energy per residue to p38α resulted from a MMGBSA calculation. Blue bars represent each residue contribution to the binding free energy of the peptide-1 p38α complex. Green bars show the van der Waals contribution to the interaction energy.

We also computed the interaction free energy between peptide-1 and p38α using the MMGBSA methodology [24] as implemented in AMBER12 [16]. This methodology allows the breakdown of the binding free energy in its different components per residue. The results of this study reveal that peptide-1 residues Asn^3^ and His^4^ are those that contribute the most to the binding free energy, and together Tyr^1^ exhibit the largest van der Waals contribution (Fig 6). More specifically, MMGBSA pairwise analysis reveals that p38α residues Leu^171^ and Val^183^ are responsible for the van der Waals interaction with Asn^3^, while Asp^177^, Glu^178^ and Thr^180^ are responsible for the van der Waals interaction with the side chain of His^4^.

**Fig 7:**
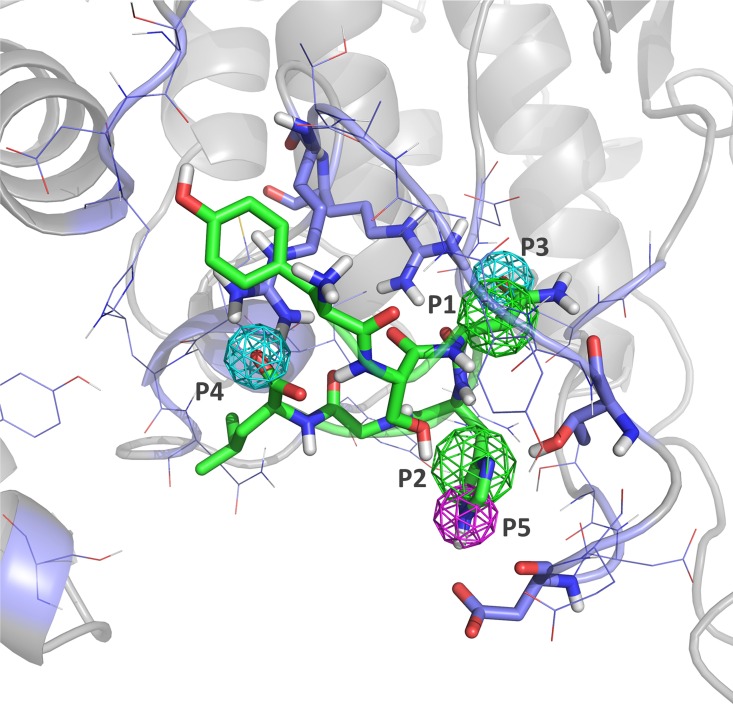
Pharmacophore of the peptide 1-p38α interaction. Hydrophobic points P1 and P2 are shown in green; proton accepting points P3 and P4 are shown in cyan; and proton donor point P5 is shown in purple.

Based on the analysis of the molecular dynamics trajectory, we could characterize the interaction between peptide-1 and p38α through a five point pharmacophore defined on the peptide that includes the most relevant interactions identified (Fig 7): Point one (P1) is a hydrophobic/aromatic moiety situated on the centroid formed by C^α^, C^β^ and C^γ^ atoms of Asn^3^; point two (P2) is also a hydrophobic/aromatic moiety situated on the centroid of the of the His^4^ side chain; point three (P3) is a hydrogen bond acceptor located on the carbonyl oxygen of Asn^3^ side chain; point four (P4) is a hydrogen bond acceptor corresponding to the carboxylic oxygen of Leu^6^ interacting with Arg^186^; and point five (P5) is a hydrogen donor point, mimicking the hydrogen bond interactions observed between the NH group of the imidazole ring of His^4^ and the backbone carbonyl oxygen and side chain oxygen atoms of Asp^177^. These points fulfill specific geometrical constraints that are defined by spheres centered on each of the points with various radii to account for the tolerance in the fulfillment of each of the points. The coordinates of the points referred to an arbitrary origin and the radius (r) of the spheres in Angstroms are: P1 (43.9, 54.7 45.9), r = 1.5; P2 (47.2, 58.9, 47.8), r = 1.5; P3 (43.6, 52.9, 46.5), r = 1.2; P4 (39.0, 61.0, 49.0), r = 1.2; P5 (47.7, 60.3, 47.3), r = 1.2.

The pharmacophore described above was subsequently used for virtual screening as described in the methods section. The screening study permitted to identify around 1300 compounds fulfilling the pharmacophore requirements in ca. 4700 conformations. These molecules were subjected to a docking study that permitted to reduce the set to 452 compounds. In order to reduce further the number of hits, molecules were subjected to a diversity analysis [19]. As a result, molecules were grouped into thirty-one clusters using the Jarvis-Patrick algorithm as implemented in the MOE program [18, 22]. Those molecules with the highest binding energy computed in the docking process (London dG score function) were used as representatives for each cluster. Of the thirty-one representative molecules only eight compounds could be purchased and tested for their ability to inhibit p38α. The results of the assays showed only four of the compounds to be active, resulting in a success rate of around 50%, similar to those reported in previous studies [25].

**Fig 8:**
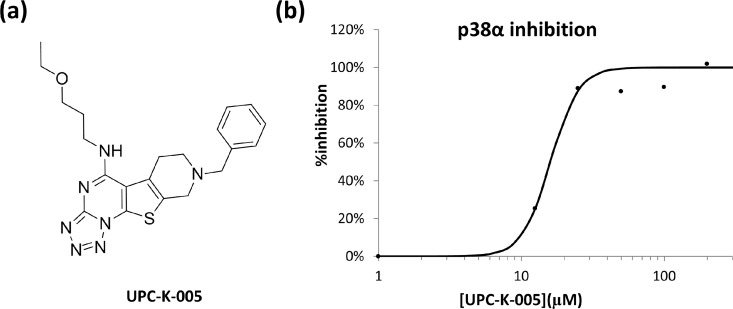
Characteristics of UPC-K-005. (a) Chemical structure of the compound UPC-K-005. (b) Dose-response inhibition curve of p38 in the presence of UPC-K-005.

The chemical structure of the most active compound discovered (UPC-K-005) is shown in Fig 8A. The compound has a tetracyclic scaffold with two legs providing a V-shape structure to the molecule. Fig 8B shows a dose-response inhibition curve of the compound performed using the ADP-Glo system with active p38α and the p38 substrate peptide (see Methods section), showing a IC_50_ of 16 μM.

In order to confirm that its phosphorylation inhibitory capability is produced through the novel site proposed, we first carried out competition experiments with ATP to demonstrate the compound behaves as a non-competitive inhibitor and second, we measured its binding affinity to two p38α mutants.

ATP competition experiments were carried out by measuring the inhibitory capability of UPC-K-005 at different ATP concentrations (see S1 Table of the supplementary material). The estimated Km for ATP for the control experiment in the absence of UPC-K-005 calculated from a Lineweaver-Burk plot is 56μM and Vmax 57788AU (arbitrary units). On the other hand, when UPC-K-005 is present at concentrations of 15 or 25μM, measured Km is 42 and 51μM, respectively with Vmax decreasing a 43% and 29%, respectively. In contrast, when the same experiment is carried out in the presence of the ATP competitive inhibitor SB203580 at 20nM [26], Km rises to 194μM without a significant change of Vmax. These results indicate that the compound acts as a non-competitive inhibitor.

We also compared the inhibitory capability of UPC-K-005 to p38αWT and to the Arg^186^Ala and Arg^189^Ala p38α mutants. The results show a sharp decrease of inhibitory capability, with a 65% inhibition decrease in the case of the former mutant and 72% inhibition decrease to the latter. For comparison reasons, we also measured the inhibitory capability of ATP competitive inhibitor SB203580 [26], showing no significant difference in its inhibitory capability between the wild type and the mutants.

**Fig 9:**
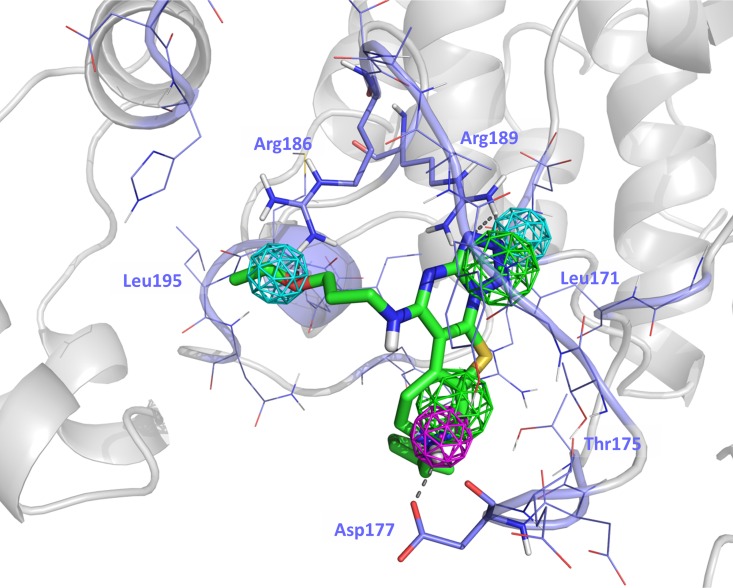
Docking of UPC-K-005 to the putative novel allosteric inhibitory site showing the fulfillment of the compound to the pharmacophore.

To understand the features of the ligand-protein interaction we carried out a docking study of UPC-K-005 to p38α. Fig 9 shows pictorially the proposed bound conformation of UPC-K-005 showing the fulfillment of the pharmacophore defined above. Specifically, the hydrophobic/aromatic moiety of pharmacophore point P1 is occupied by the tetrazole ring. Actually, it occupies a hydrophobic binding area facing Leu^171^ and Val^183^. On the other hand, the tetrahydropyridine ring of the molecule occupies the hydrophobic/aromatic moiety of point P2, being the ring positioned in the vicinity of the Asp^177^ and Thr^180^. Pharmacophore point P3 is occupied by the nitrogen of the tetrazole ring in position 2, acting as proton acceptor moiety and exhibiting a hydrogen bond interaction with Arg^189^, which also interacts though a positive charge-π interactions with the ring. Furthermore, the ether oxygen occupies the pharmacophore point P4, showing a hydrogen bond interaction with the guanidine group of Arg^186^. Finally, due to its pKa (~11) the tertiary tetrahydropyridine ring nitrogen will be protonated in aqueous solution [27], so that the proton acts as hydrogen bond donor to the oxygen atoms of the backbone and side chain of p38α Asp^177^, as required to fulfill pharmacophore point P5.

## Discussion

There are numerous examples in the literature where protein-protein interactions can be effectively inhibited using peptide sequences inspired by contact regions of the protein complex [28]. This is also true in MAPKs, where diverse peptides have been described in the literature as phosphorylation inhibitors [29]. Specifically, conserved 20–30 residue fragments at the N- or C-terminus of diverse substrates known as D- or kinase interaction motifs (KIMs) show inhibitory activity of MAPKs downstream cascades at micromolar concentrations. Their inhibitory capability is produced by binding to the so-called docking groove in the C-lobe of the MAPKs and the adjacent common docking (CD) domain and the glutamate-aspartate site (ED) [29]. The interaction between the D-motif of MK2 and the docking groove and CD/ED domains of p38α is one of the five discontinuous contact regions identified in the crystal structures of the p38α-MK2 complex. Binding studies show this contact region to be key for a tight binding affinity between the two proteins [8]. Analysis of the structure of the heterodimer permits to identify the D-motif located at the C-terminus of MK2, comprising residues Asp^366^-Ala^390^, bound to the hydrophobic docking groove of p38α, the CD domain involving residues Asp^313^, Asp^315^, and Asp^316^ and the ED site involving residues Glu^160^-Asp^161^. As mentioned above, the peptide corresponding to the fragment 370–400 of MK2 has been shown to be a potent inhibitor of p38α-dependent phosphorylation of MK2 and ATF-2, suggesting that the inhibitory capability of these D-motifs is not limited to block a specific substrate only [8]. Interestingly, due to protein flexibility the docking interaction has the ability to allosterically modulate other regions of the protein. Thus, it has been recently shown that binding of the D-motif enhances binding of ATP and in turn the catalytic activity over non docking dependent substrates, showing how these inhibitors can modulate distant regions in the protein [30].

Present results show evidence of the inhibitory profile of peptide-1 and -2 in the phosphorylation of MEF2A. As mentioned before, both peptides are segments of the MK2 regulatory loop in contact with p38α and identified after the analysis of the crystallographic structure of the MK2-p38α complex [10]. Based on modelling studies performed in this work and crystallographic evidence on similar peptides bound to the same regulatory phosphorylation region in the insulin kinase domain [12], it is expected that these peptides bind to the same site the MK2 regulatory loop does. In a further step, this modeling study was used to define a pharmacophore of the ligand-receptor interaction that was used to identify small molecule peptidomimetics. We report in the present work evidence of the inhibitory profile of UPC-K-005 in the p38α substrate peptide mediated phosphorylation *in vitro* assay and furthermore, show evidence that the compound is not an ATP-competitive inhibitor and that binds to the same regulatory phosphorylation region of p38α, based on the decrease of its inhibitory potency shown with the Arg^186^Ala and Arg^189^Ala p38α mutants.

Despite the small molecules discovered were designed to mimic peptide-1, experimental results show a differential inhibitory behavior between the peptides and UPC-K-005. Whereas peptides inhibit p38α phosphorylation in a dose-response form when MEF2A is used as substrate, they fail to inhibit p38α phosphorylation when the p38α peptide substrate is used. In contrast, UPC-K-005 inhibits both the p38α peptide substrate as well as ATF2 protein substrate in a dose-response manner, suggesting that they are more universal inhibitors. This intriguing behavior suggests that peptides and small molecules, in spite of binding to the same putative binding site they may inhibit phosphorylation through different mechanisms. A plausible explanation for this differential behavior is that peptides act by blocking the binding of a substrate protein, whereas small molecules may induce a conformational change in p38α forcing the protein to be in an inactive state. In other words, the former inhibit phosphorylation through a protein-protein inhibition disruption mechanism, whereas the small molecules may inhibit phosphorylation through an allosteric mechanism that stabilizes p38α in an inactive conformation. This is an interesting hypothesis that should be considered as preliminary and requires further investigation currently being carried out in our laboratory.

## Conclusions

We describe in this work a novel allosteric binding site on p38α that corresponds to one of the contact regions of the p38α-MK2 heterodimer where p38α is not activated. The two proteins interact at this site through two interconnected loops stabilized by several hydrogen bonds including an anti-parallel β-sheet. In order to check the functional profile of the site, we first tested the inhibitory capability of two amino acid fragments of the MK2 sequence. The results of the test show the peptides with an inhibitory capability of p38α at the micromolar range. We further identified the key interactions between the two segments through a short molecular dynamics and defined a pharmacophore for the interaction defined by five points: two proton accepting groups, a hydrogen donor group and two aromatic/hydrophobic groups. This pharmacophore was used to carry out a virtual screening process using the MOE lead-like database. The results of these studies permitted to identify a few small molecule inhibitors of p38α. The most active (UPC-K-005) exhibits a IC_50_ of 13 μM and is disclosed in the present work. In order to test that the compound binds to the new proposed site, we measured the phosphorylation inhibitory capability of UPC-K-005 on the p38α mutants Arg^186^Ala and Arg^189^Ala. The results clearly show a lower inhibitory capability in comparison to the wild type. In parallel the inhibitory capability of SB203580, known to bind to a different site [26] was measured. The results showed no difference in the inhibition capability of the compound between the wild type and the two mutants, reinforcing the hypothesis that UPC-K-005 binds to the novel site proposed.

## Supporting Information

S1 Table ATP competition experiments. p38α activity measurements and normalization at diverse concentrations of ATP in the presence of compound UPC-K-005. SB203580 is also studied for comparison purposes.
(DOCX)
