# Supplementary material for: Identification of a Novel Inhibitory Allosteric Site in p38α
Source: PLoS One. 2016 Nov 29;11(11):e0167379. doi: 10.1371/journal.pone.0167379 (PMC5127581; doi:10.1371/journal.pone.0167379)
Supplement: S1 Table — p38α activity measurements and normalization at diverse concentrations of ATP in the presence of compound UPC-K-005. SB203580 is also studied for comparison purposes. (DOCX) [file pone.0167379.s001.docx]

**Identification of a Novel Inhibitory Allosteric Site in p38α**

**Patricia Gomez-Gutierrez^1,2^, Pedro M. Campos^1^, Miguel Vega^1^ and Juan J. Perez^2^**

**^1^Allinky Biopharma. Madrid Scientific Park. Faraday, 7. Campus de Cantoblanco. 28049 Madrid, Spain**

**^2^Dept. of Chemical Engineering. Universitat Politecnica de Catalunya. ETSEIB. Av. Diagonal, 647; 08028 Barcelona, Spain**

**SUPPEMENTARY MATERIAL**

**Table S1. ATP competition experiments.** p38α activity measurements and normalization at diverse concentrations of ATP in the presence of compound UPC-K-005. SB203580 is also studied for comparison purposes.

**
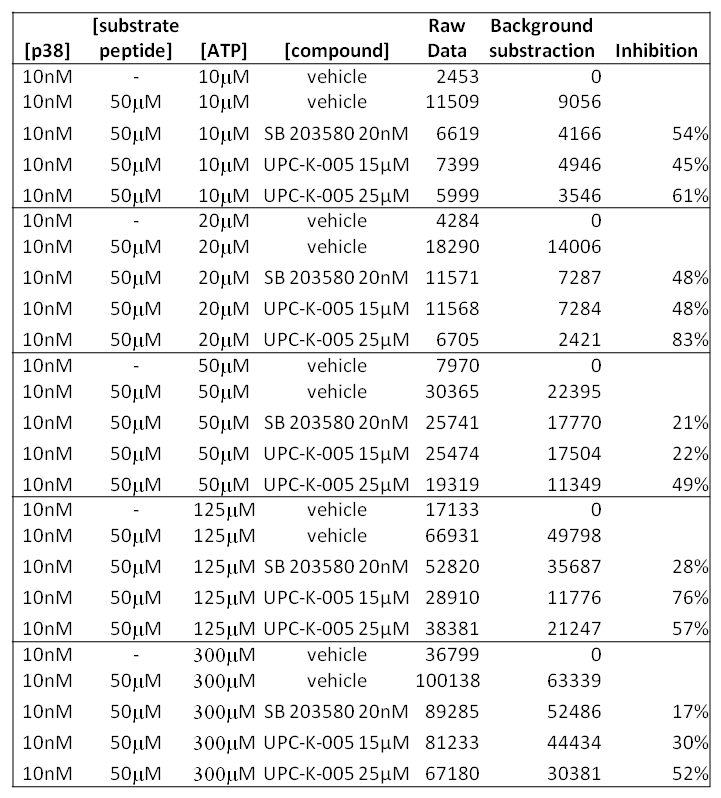
**
